# The Relationship of Social Media Addiction With Internet Use and Perceived Health: The Moderating Effects of Regular Exercise Intervention

**DOI:** 10.3389/fpubh.2022.854532

**Published:** 2022-05-06

**Authors:** Bo-Ching Chen, Mei-Yen Chen, Yu-Feng Wu, Yu-Tai Wu

**Affiliations:** ^1^Physical Education Research and Development Center, National Taiwan Normal University, Taipei City, Taiwan; ^2^Graduate Institute of Sport, Leisure and Hospitality Management, National Taiwan Normal University, Taipei City, Taiwan; ^3^Office of Physical Education, Ming Chi University of Technology, New Taipei City, Taiwan; ^4^Office of Physical Education, Soochow University, Taipei City, Taiwan

**Keywords:** internet addiction disorder, social networking sites, physical activity, perceived health, public health

## Abstract

The popularity of online social media in recent years has not only brought information and social convenience to people's lives, but has also given rise to many problems, among which social media addiction (SMA) has become a concern of many scholars and experts. Past research has shown that regular exercise (REx) can have many health benefits for the body, so numerous scholars and experts believe that this may be one possible strategy for reducing the health effects of online community addiction and Internet use (IU). Therefore, this study adopted a secondary data research approach to explore and predict the effect of age on social media use and personal health, and therefore included age as a control variable to investigate whether the intervention of REx, excluding the effect of age, moderates the effect of SMA on IU and on perceived health (PH). The participants of this study were adults aged 18 years or older in Taiwan, using the 2019 “Survey Research Data Archive,” Vol. 7, No. 5 data. A total of 1,933 questionnaires were retrieved, and after elimination of invalid responses, 1,163 data were analyzed using Partial Least Squares Structural Equation Modeling, PLS-SEM. The results were as follows: (1) SMA positively affected IU, (2) SMA could negatively affect PH, (3) there was no statistical effect of IU on PH, (4) SMA did not indirectly affect PH through IU, (5) REx had a moderating effect on SMA and IU, and (6) REx did not regulate the effect of SMA on PH. First, from these results, it is clear that the negative health effects of SMA may not be simply due to prolonged IU. Secondly, while it is true that the moderating effect for people with low levels of SMA can reduce IU, for people with high levels of SMA, the moderating effect of REx becomes a catalyst for increased Internet usage behavior. Finally, we draw conclusions based on the results of the study and propose directions and recommendations for follow-up research.

## Introduction

In the Web 2.0 era, websites have evolved from passive browsing to a sharing and participation framework, and the focus of website management has shifted to community development. Traditionally, a community is defined as a group of people who are willing to get together or take on a common responsibility (1). Today's social media are platforms created in the virtual world of the Internet which allow users to freely create their own unique accounts, discuss, share, and exchange information in this cyberspace via text, images, audio, and video (2, 3). The rise of online social media has thus created a different mode of interpersonal interaction than traditional ones, and the use of social media sites has become one of the most popular social behaviors today (4). The most common social media in the world today are Facebook, Instagram, Twitter, and Snapchat (5). Taiwan's use of social media is also similar to that of other countries. In the Taiwan Internet Report in 2020, the top three social media platforms were Facebook (94.2%), Instagram (39.2%), and Twitter (6.4%) (6).

The hierarchy of needs theory (Maslow's hierarchy of needs) (7) suggests that people have social needs, and one of the key factors affecting the basic need for emotion and belonging is interpersonal communication (8). One of the main functions of social media is to build interpersonal interaction networks of personal relationship resources (9); therefore, the use of social media can bring users a sense of social connection, access to information, enhancement of their emotions, and motivation (10, 11), it can improve self-esteem and quality of life (12), and it can even inspire people to lead social movements (13). The use of online communities has become an important part of daily life (14, 15).

Although social media are an important window into the outside world (16) and provide many conveniences for networking and social relationship building, the use of social media can also have many negative effects (10). Social media addiction (SMA) is the result of excessive use of the Internet. Studies have found that SMA is associated with increased use of electronic devices, cell phones, the Internet (17–20), more sedentary time (20), poor sleep quality (21, 22), lower physical activity (17, 18, 23, 24), rise in BMI value (25), negative health effects (19, 26), poor mental health status (e.g., anxiety and depression) (22, 25), distant family relationships (17), and low academic performance (12, 25). Therefore, SMA has a great impact on individuals' physiology, psychology, and life, and online SMA has become a serious health problem for people around the world (9, 17, 27).

Regular exercise (REx) or continuous physical activity can have many health benefits for the body, such as improving sleep quality, improving executive brain function, reducing symptoms of depression, reducing anxiety, improving the quality of people's daily lives, and improving the physical function of people of all ages so they can live each day energetically (28). In addition to the health benefits of REx, previous studies have found a negative relationship between social network addiction and REx (17, 18, 23). Improving social network addiction is difficult, as medication may have side effects that affect health, and psychotherapy is difficult to design for specific cases (29). This is why REx has been considered by many to be one of the more viable options for improving people's online social media addiction (29–32).

Although scholars believe that exercise is one of the possible solutions to improve SMA, there is relatively little empirical research and little attention has been paid to how the use of REx affects SMA and its impact on perceived health (PH) (17, 20, 26, 33). In addition, previous studies on social media addiction has less concern for adults in the general society. However, in fact, most teenagers are still students in school, their social circles are homogeneous, and the frequency and time of social media use by minors are subject to a certain degree of control and supervision by parents or school teachers (34, 35). On the contrary, adults are not subject to the above restrictions, have a wider social circle, and use the Internet more freely, so there is still a need to pay attention to the problem of adult Internet addiction. Finally, based on the above, the purpose of this study is to investigate whether REx among adults in general society moderates the relationship between SMA, Internet use (IU), and PH.

### Social Media Addiction

Social media have emerged as a new application due to the development of Internet technology, so in order to understand SMA, we need to start by understanding internet addiction. After about 1995, the topic of Internet addiction began to be noticed by scholars and experts, mostly from the traditional viewpoint of addictive disorders. Goldberg (36) was one of the first scholars to focus on this phenomenon. He used the term internet addiction disorder and proposed seven criteria for determining it, which led to the common use of the term “addiction” in subsequent studies. However, some scholars have begun to reflect that the use of the word “addiction” tends to give the impression of being labeled as a disease and creates prejudice (37). Therefore, some scholars use other similar terms such as pathological IU (38–40), problematic IU (41–43), and compulsive IU (44, 45).

Due to the many related terms mentioned above, and in order to avoid confusion, this study uses the more common term SMA, with reference to Diagnostic and Statistical Manual of Mental Disorders (DSM-IV) measures of substance or behavioral addiction and characteristics of social media overuse (46–48). From the health, psychological and behavioral aspects of the general public were selected to better fit the definition of this study, SMA was defined as a variety of adverse physical and psychological reactions or inappropriate social behaviors resulting from excessive use of various types of social networking media by community members.

### Internet Use

Social media allow users to freely socialize, circulate information, entertain, spend time, relax, make friends, pay attention, learn about others, or seek status for themselves on the Internet (4, 49, 50). Due to the wide range of network usage, researchers have proposed the Technology Acceptance Model, TAM, in the field of information systems (51–53), which has been applied to related studies such as social media usage by medical professionals (54) and Facebook usage behavior (55). Therefore, IU in this study refers to the use of Internet-mediated services or behaviors that users need to apply, whether at work or leisure, for example, using social networking sites, communication software, buying and selling things online, doing business or working, playing games together, and sharing videos.

### Perceived Health

The World Health Organization (56) defines health as not only the absence of disease, but also the physical, psychological, and social integrity and well-being of the individual. However, in epidemiological and gerontological studies, objective physiological and biochemical indicators were traditionally used to assess the health of individuals until 1982, when scholars Mossey and Shapiro (57) proposed a similar approach to self-assessment of health. For example, how do you feel about your health compared to people of the same age? Or how would you rate your health? This self-perceived level of health is used to predict mortality in older people. Although self-conscious health measures may not be as accurate as objective physiological and biochemical indicators, they do provide an alternative and more convenient way to assess one's health status. This method is still widely used in many research studies (58–60). Therefore, PH in this study mainly refers to the individual's awareness of their physical health status, which includes subjective and objective evaluation of their own physical and mental health, and adopts the viewpoint of self-comparison of changes in their health at different points in time.

### Regular Exercise

It is well-known that regular physical activity (exercise) is beneficial for physical and mental health (61, 62), reduces the risk of many chronic diseases such as hyperglycemia and lipids (63), promotes social engagement and improves self-estimated well-being (64), and can increase health literacy, develop healthy lifestyles, and improve obesity and depression-related problems (65). Therefore, REx is an important key factor in the prevention of many diseases (66). Current recommendations for weekly exercise state that all healthy adults over the age of 18 should have at least 150–300 min of moderate-intensity aerobic exercise or 75–150 min of high-intensity aerobic exercise per week (67). In addition, weight training should be performed at least 2 days per week to maintain or increase muscle strength and endurance. REx in this study refers to a regular number of exercises per week that has been maintained for a period of time.

## Research Methods

### Research Hypotheses

#### The Relationship Between Social Media Addiction, Internet Use, and Perceived Health

According to the definition of health by The World Health Organization (56), we can divide health into psychological and physiological aspects. Previous studies have found health effects of social media addiction such as: poor mental health (e.g., anxiety and depression) (22, 25), fear of missing out (FOMO) (68), which are among the effects on psychological health dimensions (69–71). The physiological health effects of social media addiction (SMA) are partially associated with prolonged Internet use (17–20), such as: decreased physical activity (17, 18, 20, 23, 24), e.g., more sedentary time (20). In other words, there are two main ways in which social media addiction affects health: one directly affects mental health, and the other affects physical health through outward Internet use.

Some scholars believe that excessive use of social media, or even compulsive-like social media use, in such extreme situations may produce similar results to those of past traditional substance addictions (68). While social media users can be divided into two types according to their behaviors: active participation and passive participation (72). Active participation refers to behaviors that enhance communication and information generation, such as status updates, check-ins, messages, and so on. Passive participation refers to passive information browsing behavior that lacks the purpose of communication, such as viewing information on the Internet and browsing information in friends' circles (72). In order to make the social network more active, the overall design of social media platforms is to allow users to share continuously. In order to attract the attention of others and increase the exposure or viewing rate among friends, users will inevitably need to like, update posts, check-in, and create stories from time to time, and increase the richness and topicality of issues in the community through their active sharing behavior. In other words, the entire social media framework is based on the sharing and participation behavior of users (73).

According to Pervin (74) Person Environment fit theory, which emphasizes the degree of fit between individuals and their environment, the higher the interaction between people and their environment, the higher the satisfaction and the lower the stress in life. From this theoretical point of view, we can consider online social media as a virtual society. When social users want to integrate and match this social media world, they are bound to behave in accordance with the rules and expectations of this environment. When people with a high level of SMA want to be more integrated, they will engage in more behaviors than the average person, regardless of the type of engagement behavior (active, passive) or the type of device (phone, tablet, or computer) they use, which is ultimately reflected in their external use of the Internet. When using the Internet for too long, it is most often accompanied by health problems such as eye fatigue, anger, and sleep disorders (75). Sedentary and physically inactive lifestyles have many negative health effects (20). Therefore, this study suggests that there are two pathways by which SMA affects PH. The higher the level of SMA, in addition to the original effect on mental health; social media users increase their online behavior to adapt to the social media environment to which they belong, and this process in turn has an effect on physical health. Based on the above, the hypotheses are proposed as follows:

H_1_: Social media addiction has an effect on Internet use.
H_2_: Social media addiction has an effect on perceived health.
H_3_: Internet use has an effect on perceived health
H_4_: Social media addiction has an indirect effect on perceived health through Internet use.

#### The Moderating Effect of Regular Exercise Intervention

Many studies have confirmed the negative relationship between regular exercise and SMA (17, 18, 23). In the psychological perspective, social media addiction is often accompanied by impulsivity, depression, anxiety, and obsessive-compulsive disorder (76). In addition to the physical benefits of exercise, Csikszentmihalyi (77) proposed the idea of “flow experience,” which means that when the participant concentrates on the activity, it will produce a subjective and temporary experience of multiple feelings such as oblivion, smoothness, and joy (78–80). Therefore, exercise can reduce depression, anxiety, and anger, and improve mood (81).

From the physiological perspective, neurobiological and neuropsychological studies found that Internet addiction leads to neurostructural changes that reduce the activity of the dopaminergic system and limit neurocognitive function, whereas regular exercise can reduce SMA by moderating the central and autonomic nervous systems (29). It is clear from the above that exercise can improve SMA and promote health through many different biopsychological mechanisms. Therefore, many scholars believe that exercise can improve the symptoms associated with social media addiction (29–32).

Based on this, we can imagine that the more people exercise regularly each week, the less time and opportunities they have to use the Internet than people who do not exercise regularly; the many benefits of regular exercise, such as improved psychological and physiological health, may also change the impact of SMA on PH. Therefore, this study hypothesized that regular exercise could moderate the effects of SMA on IU and SMA on PH. Based on this, the hypotheses of this study are as follows:

H_5_: Regular exercise moderates the effect of social media addiction on Internet use.

H_6_: Regular exercise moderates the effect of social media addiction on perceived health.

#### Effect of Age

Many factors are known to influence social media addiction and health, such as physical factors (age, gender), social factors (frequency of use, need for social media), and psychological factors (stress, empathy, responsibility, depression, and social support) (82). This study was conducted with adults over the age of 18, spanning multiple groups such as young people, young adults, and older adults. It is known from previous studies that the older a person is, the more likely he or she is to have health problems, and the older the age, the less the Internet use (83, 84). Therefore, in order to reduce the bias caused by different generations, age was used as a control variable in this study to control for the effect of age in order to obtain a result excluding the effect of age.

### Research Model

**Figure 1:**
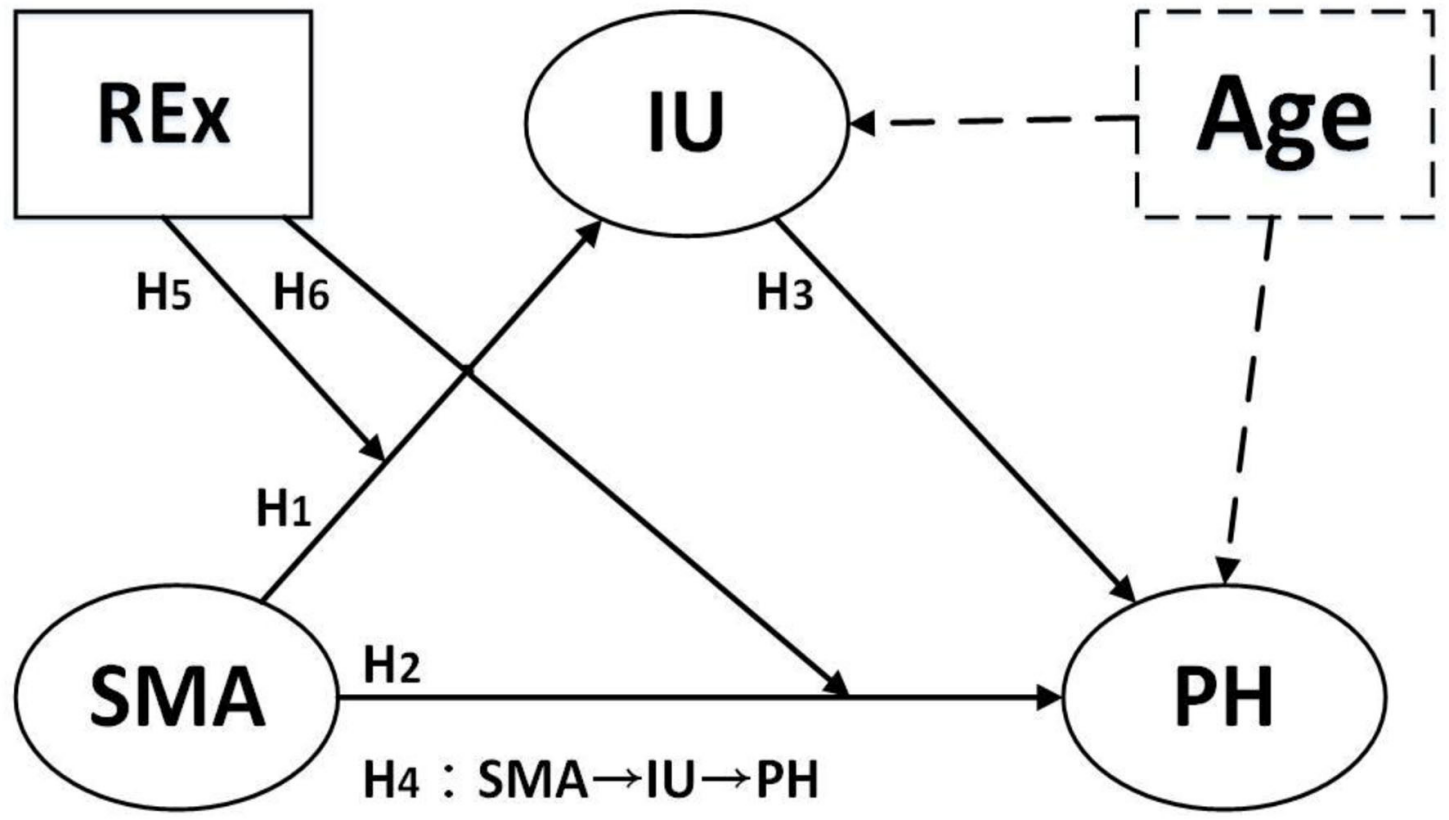
Research model. Age is a controlled variable.

Based on the theoretical framework of hierarchy of needs theory (7) and the person environment fit theory (74), this study constructed a research model. First, this study included “age” as a control variable, and then after excluding the effect of age, the hypothesized relationships among the variables SMA, IU, and PH were tested. Second, the moderating effect of the interaction between REx and SMA by using REx to moderate the relationship between SMA on IU and PH was tested. This study proposed six hypotheses and constructed a research model which can be seen in Figure 1.

### Research Procedure

The Taiwan Social Change Survey (TSCS) in Taiwan is an annual survey conducted by the Ministry of Science and Technology since 1990, in which two independent questionnaires are conducted, in which a rigorous sampling is conducted on the current distribution of Taiwan's population, with very diverse issues, such as technology and risk, social inequality, work and life, religion, health, and leisure life. Up to now, 66 surveys have been accumulated, and the data obtained have been provided to the academic community for research and analysis at no cost. In the 2019, Taiwan Social Change Survey (Round 7, Year 5): Technology and Risk Society (85), the data are consistent with the topic of this study. The TSCS is a very rigorous sampling and distribution of samples throughout Taiwan make it very suitable for secondary data researchers to conduct research and analysis. Therefore, this study conducted a secondary data analysis.

The survey data used in this study are based on the population of adults aged 18 years or older in Taiwan, and the population ratios of each stratum were projected from July 2019 to February 2020 based on the 2018 demographic data of the Department of Household Registration, using a stratified three-stage sampling method. Except for the East Taiwan region, where a two-stage sampling method was adopted due to the large difference in population distribution, in all other regions, the first tier was drawn for “rural urban areas,” followed by “villages,” and finally “people,” so that each person in the parent body had a non-zero chance of being selected, and each selected case was irreplaceable. A total of 1,933 questionnaires were successfully completed in a total of 130 villages sampled throughout Taiwan.

### Measurements

As mentioned earlier, SMA, IU, and PH are the three main variables of this study. “Age” was used as a control variable in the demographic variables, and after excluding the effect of age, “REx” was used as a moderating variable to investigate the exercise intervention. The summary of each component variable is shown in Table 2.

#### Age

As a control variable to exclude the effect of “age” in this study, the question was: “When were you born? (year, month, and day).” The value calculated by subtracting the year of completion from 2019 was used to represent the age of the respondent at the time of completing the questionnaire, with larger values representing older respondents. The age range of respondents in this study was between 18 and 81 years old.

#### Regular Exercise

This study focused on the moderating variables of the exercise intervention, using the questions: “Have you exercised consistently or regularly in the last 3 months? If yes, how many times per week on average?” The higher the number of times means the more the respondents exercise per week. Respondents in this study answered from 0 to a maximum of 15 times per week for “no continuance or REx.”

#### Social Media Addiction

For this study, the SMA scale was adapted from (9, 46) with the SMA variable as the main predictor variable in this study, consisting of five questionnaire items to identify the psychological distress caused by social media addiction. The main question asked respondents to recall if they had experienced the following five scenarios in the past 12 months: “I continuously want to use social networking sites or communication software,” “I use social networking sites or communication software to forget my personal problems,” “I have tried to reduce the time I spend on social networking sites or communication software, but I have not succeeded,” “I feel confused or agitated when I can't use social networking sites or communication software,” or “I use social networking sites or communication software too often, which may have a negative impact on my studies/work.” The measurement is based on a 5-point Likert scale (1 = *very non-compliant*, 2 = *mostly non-compliant*, 3 = *partially non-compliant*, 4 = *partially compliant*, 5 = *mostly compliant*, and 6 = *very compliant*), and the higher the response value, the more addicted the community is to the network.

#### Internet Use

For this study, the IU scale was adapted from (54) and (55). The IU variable was used as the mediator variable in this study, consisting of three questions to identify observable behaviors of daily Internet use among individuals. Question 1: “In the past year, how often did you use social networking sites or communication software (e.g., Facebook, blogs, YouTube, Line, Skype, WeChat, etc.) to chat, communicate, play games, or share videos with others?” The other two questions were: “I often use the Internet to buy and sell things, do business or work” and “I often use communication software at work.” The measurement questions were on a 6-point Likert scale, and the order of the original questionnaire (1 = *several times a day* ~ 6 = *none of them*) was reordered as: “1 = *none of them*, 2 = *once a month or less*, 3 = two *or three times a month*, 4 = two *or three times a week*, 5 = *almost every day*, and 6 = *several times a day*,” where the higher the score, the more often the respondent uses the Internet.

#### Perceived Health

Mossey and Shapiro (57) proposed the use of a similar self-rated health approach to predict health. In this study, PH was measured by combining physical and psychological aspects and by self-comparing the changes of PH at different time points.

The PH variable is the outcome variable of the main model in this study. It is a combination of three observation questions in which respondents were asked to compare changes in their “physical health status,” “mental/spiritual health status,” and “sleep status” over the past year. A 5-point scale was used for the questions (1 = *much worse*, 2 = *a little worse*, 3 = *no change*, 4 = *a little better*, and 5 = *much better*). The higher the score, the better the PH status.

### Pre-test

In this study, 1,933 questionnaires were received and after rigorously removing 659 invalid data (missing values, skipped answers), 1,274 valid data remained.

This study was conducted using the secondary data research method in two stages, starting with a small portion of data 111 (10%) data in IBM SPSS 24.0 for Windows. We then verified that the questions were within the designed domains as expected. In the second stage, the remaining 1,163 (90%) data were verified with Smart PLS, a more rigorous structural equation modeling (SEM) with confirmatory factor analysis (CFA), to verify the hypotheses model, mediation effects, and moderation effects. The results of the item analysis, reliability analysis, and exploratory factor analysis of the 111 samples in the pre-test were analyzed as follows.

#### Item Analysis

For SMA, five questions had critical ratio (CR) values ranging from 6.76 to 11.10; for IU, three questions had CR values ranging from 5.55 to 8.45; and for PH, three questions had CR values ranging from 6.40 to 8.19. All questions had a significant CR-value > 1.96, which means that each question in this study had good item discrimination (for details, see Table 1).

**Table 1:** Item and reliability analysis (*N* = 111).

**Construct**	**Item analysis**			**Reliability analysis**		
**Category**	**Frequency (** ***M*** **)**	**Average difference**	**CR**	**Correlation**	**Corrected item-total correlation**	**α**
1. SMA							
High	35 (2.89~4.97)	1.67~3.00	6.76~11.10	0.24~0.54	0.46~0.56	0.75
Low	37 (1.16~1.97)					
2. IU							
High	43 (5.00~5.72)	1.21~2.81	5.55~8.45	0.31~0.52	0.44~0.55	0.69
Low	41 (2.20~4.51)					
3. PH							
High	61 (3.13~3.31)	0.77~1.03	6.40~8.19	0.46~0.64	0.63~0.78	0.77
Low	50 (2.10~2.54)					

#### Reliability Analysis

DeVellis and Thorpe (86) stated that Cronbach's alpha is recommended to be unacceptable when it is below 0.60, the Cronbach's α values for SMA, IU, and PH were 0.75, 0.69, and 0.77; which is >0.6 recommended standards. According to (87), a relationship between items should be >0.3. The correlation between the items SMA2 and SMA5 in SMA was 0.24 which was slightly lower than the standard. However, the correlation between the questions in all other items was between 0.31 and 0.64, which was in line with the recommendation of (87), that is, >0.3. In summary, the internal consistency of all the components was met in this study (see Table 1 for details).

#### Exploratory Factor Analysis

In order to verify whether the questions conformed to the expected configuration of the study and that there was no problem of excessive cross loading across the configuration, an exploratory factor analysis (EFA) was conducted with 111 samples. The results of the selected spindle factor algorithm and the Promax diagonal rotation in the Bartlett spherical check showed that the variables were correlated (**χ**^2^ = 325.96, *df* = 55, < 0.001). The KMO (Kaiser-Meyer-Olkin) sampling appropriateness value of 0.739 is in line with (88), where a KMO of between 0.7 and 0.8 is still appropriate (middling) for factor analysis, indicating the existence of common factors in this scale. The eigenvalues of 3.26, 2.11, and 1.23 were extracted using direct oblimin analysis, and three eigenvalues >1 factor were extracted. Factor 1 consists of SMA1~SMA5, a total of five questions, and the factor loadings ranged from 0.42 to 0.84, which is in line with the social media addiction construct designed in this study. Factor 2 consists of SMA1 and IU1~IU3, a total of four questions, with factor loadings ranging from 0.41 to 0.90, which is in line with the IU of this study questionnaire. Factor 3 consisted of four questions from PH1 to PH3, with negative factor loadings ranging from 0.58 to 0.83, in line with the PH of the questionnaire in this study. However, because of the cross loading between factor 1 and factor 2 for SMA1, it was deleted (for details, see Table 2).

**Table 2:** Exploratory factor analysis (*N* = 111).

**Items**	***M***	***SD***	**Construct**		
			**1.00**	**2.00**	**3.00**
SMA3	2.35	1.49	0.84		
SMA2	2.32	1.34	0.59		
SMA4	2.71	1.55	0.51		
SMA5	1.93	1.18	0.43		
SMA1	3.65	1.71	0.42	0.41	
IU2	3.95	1.92		0.90	
IU1	5.19	1.07		0.56	
IU3	3.90	2.16		0.50	
PH2	2.96	0.74			0.83
PH3	2.88	0.75			0.77
PH1	2.67	0.83			0.58
Eigenvalues			3.26	2.11	1.23
Variance explained			24.71	15.30	6.92

*Values below 0.4 are not displayed*.

## Results

After pre-testing to confirm that the constructs had good reliability and validity, the main study which consisted 1,163 data, were analyzed by PLS-SEM using Smart PLS, and the results were as follows.

### Participants (Demographic Analysis of the Main Study)

Among the 1,163 samples analyzed, 513 (44.11%) were females and 650 (55.89%) were males. To facilitate the understanding of the “age” distribution, in the descriptive statistics the results are presented as follows: 276 (23.73%) participants were 30~39 years old, 264 (22.7%) were 18~29 years old, 241 (20.72%) were 40~49 years old, 219 (18.83%) were 50~59 years old, 133 (11.44%) were 60~69 years old, and 30 (2.58%) were 70 years old or over. Participants' “REx per week” is also presented in the descriptive statistics; the most is no REx (0 times) for 584 participants, accounting for 50.21%, followed by 141 participants exercising three times per week, accounting for 12.12%, 7–9 times by 128 participants, accounting for 11.01%, 2 times by 121 participants, accounting for 10.40%, 4–6 times by 109 participants, accounting for 9.37%, once by 75 participants, accounting for 6.45%, 5 participants, or 0.43% for more than 10 times. Please refer to Table 3 for the rest of the details of the background variables: income, work, education, marital status, and place of residence.

**Table 3:** Descriptive analysis (*N* = 1,163).

**Variables**	**Participants (%)**	**Variables**	**Participants (%)**
**1. Gender**		**5. Educational level**	
(0) Female	513 (44.11)	(1) Elementary	38 (03.27)
(1) Male	650 (55.89)	(2) Middle School	100 (08.60)
**2. Age**		(3) High School	321 (27.60)
(1) 18–29	264 (22.70)	(4) College	149 (12.81)
(2) 30–39	276 (23.73)	(5) University	403 (34.65)
(3) 40–49	241 (20.72)	(6) Master	135 (11.61)
(4) 50–59	219 (18.83)	(7) Doctorate	17 (01.46)
(5) 60–69	133 (11.44)	**6. Marital status**	
(6) 70 and higher	30 (02.58)	(1) Single	407 (35.00)
**3. Monthly income (NTD)**		(2) Married	642 (55.20)
(1) 20,000 and Below	209 (17.97)	(3) Divorced	69 (05.93)
(2) 20,001~40,000	429 (36.89)	(4) Other	45 (03.87)
(3) 40,001~60,000	285 (24.51)	**7. REx per week**	
(4) 60,001~80,000	111 (09.54)	(1) 0 times	584 (50.21)
(5) 80,001~100,000	42 (03.61)	(2) 1 times	75 (06.45)
(6) 10,001 and Higher	61 (05.25)	(3) 2 times	121 (10.40)
(7) Others	26 (02.24)	(4) 3 times	141 (12.12)
**4. Occupation**		(5) 4–6 times	109 (09.37)
(1) Agriculture, Forestry, Fisheries, and Livestock	27 (02.32)	(6) 7–9 times	128 (11.01)
(2) Mining and earthwork industry	1 (00.09)	(7) 10 or more times	5 (00.43)
(3) Manufacturing Industry	268 (23.04)	**8. Current residence**	
(4) Electricity and gas supply industry	3 (00.26)	(1) Keelung City	32 (02.75)
(5) Water supply and pollution control industry	4 (00.34)	(2) Taipei City	157 (13.50)
(6) Construction industry	72 (06.19)	(3) New Taipei City	215 (18.49)
(7) Wholesale and Retail	175 (15.05)	(4) Taoyuan City	57 (04.90)
(8) Transportation and Storage	53 (04.56)	(5) Hsinchu City	23 (01.98)
(9) Accommodation and Catering	89 (07.65)	(6) Hsinchu County	49 (04.21)
(10) Information and Communication	36 (03.10)	(7) Miaoli County	72 (06.19)
(11) Finance and Insurance	43 (03.70)	(8) Taichung City	165 (14.19)
(12) Real Estate	11 (00.95)	(9) Changhua County	44 (03.78)
(13) Professional, Scientific, and Technical Services	60 (05.16)	(10) Nantou County	3 (00.26)
(14) Service Support	42 (03.61)	(11) Yunlin County	15 (01.29)
(15) Public Administration and National Defense; Mandatory Social Security	52 (04.47)	(12) Chiayi City	1 (00.09)
(16) Education Services	78 (06.71)	(13) Chiayi County	5 (00.43)
(17) Health Insurance and Social Work Services	57 (04.90)	(14) Tainan City	96 (08.25)
(18) Arts, Entertainment and Leisure Services	24 (02.06)	(15) Kaohsiung City	121 (10.40)
(19) Other Service Industry	64 (05.50)	(16) Pingtung County	16 (01.38)
(20) Other	4 (00.40)	(18) Hualian County	31 (02.67)
		(19) Yilan County	38 (03.27)
		(20) Penghu County	9 (00.77)
		(21) Other	14 (01.20)

Finally, the remaining 1,163 samples, about 90%, were analyzed by partial least squares structural equation modeling (PLS-SEM) for subsequent validation.

After the above steps were verified, the SMA component was finally identified as SMA2~SMA5, with four questions, and the IU component IU1~IU3, with three questions. The PH, PH1~PH3, consisted of three questions; this formed the source of the subsequent PLS-SEM analysis.

### Construct Reliability and Validity Analysis

Structural equation modeling analysis was suggested by scholars (89) as a two-step analysis. The first stage is to verify that the measurement model is ideal for each potential structure, and then the second stage is to evaluate the structural model. The study was conducted after confirming the overall model before the mediation and adjustment validation. In this study, Smart PLS software was used, and according to scholars such as (90) and (87), a good measurement model needs to meet the following requirements: (1) the standardized factor loadings are >0.6; (2) the composite reliability is >0.7; and (3) the average variance extraction is >0.5. When the above criteria are met, it means that each component has convergent validity. This study measured: (1) SMA, (2) IU, (3) PH, (4) age, and (5) number of exercises per week, that is, five variables with 12 questions in total. The results of the analysis showed that the factor loadings of each question ranged from 0.60 to 0.88, all of which were >0.6; the reliability of the components ranged from 0.80 to 0.82, which were all >0.7; and the mean variable extractions ranged from 0.53 to 0.61, which were all >0.5, which met the criteria suggested by academic experts. In other words, all the variables in this study had convergent validity (see Table 4 for details).

**Table 4:** Construct reliability and validity analysis.

**Construct**	**Items**	**Min/Max**	***M(SD)***	**Kurtosis**	**Skewness**	***FL***	***t***	***CR***	**AVE**
**1. SMA**									
	SMA2	1/6	2.29 (1.40)	−0.09	0.95	0.75	32.22	0.82	0.53
	SMA3	1/6	2.48 (1.47)	−0.61	0.70	0.72	28.21		
	SMA4	1/6	2.62 (1.60)	−0.85	0.61	0.76	33.17		
	SMA5	1/6	1.94 (1.23)	0.84	1.29	0.67	24.70		
**2. IU**									
	IU1	1/6	5.37 (1.01)	5.95	−2.28	0.62	17.58	0.80	0.57
	IU2	1/6	4.14 (1.87)	−1.17	−0.57	0.85	64.75		
	IU3	1/6	4.23 (2.03)	−1.12	−0.77	0.78	37.09		
**3. Perceived health**									
	PH1	1/5	2.73 (0.83)	0.09	0.46	0.88	26.53	0.82	0.61
	PH2	1/5	2.97 (0.76)	0.58	0.24	0.84	30.59		
	PH3	1/5	2.77 (0.74)	0.71	−0.02	0.60	8.66		
**4. Controlled variable**									
	AGE	18/81	42.27 (14.37)	−0.85	0.26	1.00	–	1.00	1.00
**5. Moderating variable**									
	REx	0/15	1.90 (2.48)	0.96	1.25	1.00	–	1.00	1.00

### Construct Discriminant Validity

Discriminant validity analysis was conducted to verify whether the statistical differences between the different constructs were achieved by using the AVE method. (90) proposed to use the root value of average variance extracted (AVE) for each construct. If it is larger than the two correlation coefficients of each construct, it means that the constructs have discriminant validity (see Table 5). The AVE values were between 0.73 and 0.78, and the two correlations between the constructs were between −0.39 and 0.29. The minimum value of AVE was 0.73, which is greater than the maximum correlation between the constructs (−0.39). Therefore, there was good discriminant validity between the constructs of this study.

**Table 5:** Construct discriminate analysis.

	**AVE**	**(1) SMA**	**(2) IU**	**(3) PH**	**(4) AGE**	**(5) REx**
(1) SMA	0.53	0.73				
(2) IU	0.57	0.29	0.76			
(3) PH	0.61	−0.07	0.05	0.78		
(4) AGE	1.00	−0.31	−0.39	−0.10	1	
(5) REx	1.00	−0.11	−0.11	0.12	0.02	1

### Model Verification

It was further confirmed that the variance inflation factor (VIF) was between 1.037 and 1.336 for all variables, which is less than the 3.3 criterion suggested by (91). The standardized root mean square residual (SRMR) is 0.073, which is less than the SRMR value of 0.08 as suggested by (92), which is a good criterion for model fit. In other words, it means that there was no co-linearity between the model variables in this study and the fit was good.

In the structural model verification, the hypothesis validation was divided into two models. Model 1 was to investigate the relationship between SMA, IU, and PH after excluding the influence of age. Model 2 was an adjustment model in which REx was put into the model to investigate its moderating effect. The model path coefficients and hypotheses are described as follows.

#### Model 1

**Figure 2:**
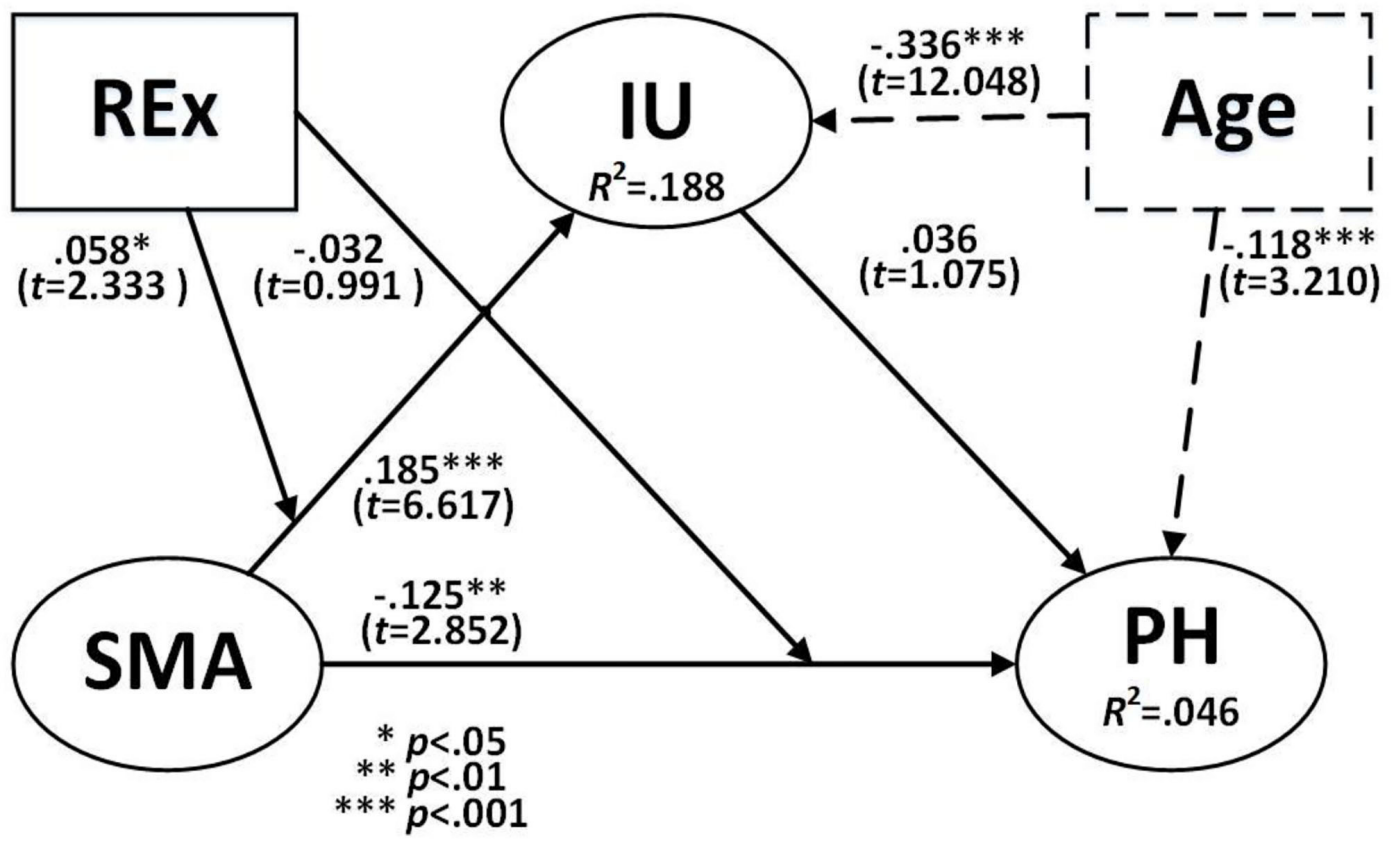
Validation of the research model. Age is a controlled variable.

Model 1 was the main model, and only the hypotheses H_1_~H_4_ between SMA, IU, and PH were tested here. First of all, in the control variables section, the standardized path coefficient of “age” for IU was −0.335, *t*-value 12.048, and *p* < 0.001 was significant. The standardized path coefficient of “age” on PH was −0.118, with a *t*-value of 3.210 and a significant *p* < 0.001. In other words, age did have a controlling effect in the model of this study. H_1_: SMA positively affects IU. The standardized regression coefficient of SMA on IU was 0.185, which was significant (*t* = 6.617, *p* < 0.001), so hypothesis 1 of this study was valid and positive. H_2_: SMA negatively affects PH. The standardized regression coefficient of SMA on PH was −0.125, which was significant (*t* = 2.852, *p* = 0.004 < 0.01), so hypothesis 2 of this study was valid and negative. H_3_: IU had no significant effect on PH. The standardized regression coefficient of IU on PH was 0.036, which was not significant (*t* = 1.075, *p* = 0.278 > 0.05); therefore, hypothesis 3 of this study was not valid. H_4_: SMA did not indirectly affect conscious health through IU. The standardized regression coefficient for the effect of SMA on PH through IU was 0.007, which did not meet the significant level (*t* = 1.048, *p* = 0.295 > 0.05), so the hypothesis 4 mediating effect of this study was not valid (see Table 6 and Figure 2).

**Table 6:** Overall structural model parameter estimation table.

**Hypotheses**	**Constructs**	**Model 1**				**Model 2**			
		**β**	***t***	***R* ^2^**	***f^**2**^***	**β**	***t***	***R* ^2^**	***f^**2**^***
H1	SMA → IU	0.185***	6.617	0.185	0.038	0.188***	6.858	0.188	0.039
H2	SMA → PH	−0.125**	2.852	0.022	0.014	−0.111***	2.939	0.046	0.011
H3	IU → PH	0.036	1.075		0.001	0.032	0.978		0.001
H4	SMA → IU → PH	0.007	1.048			0.006	0.941		
**Controlled variable**									
	AGE → IU	−0.336***	12.048		0.125	−0.336***	11.569		0.116
	AGE → PH	−0.118***	3.210		0.011	−0.168***	5.206		0.022
**Moderating effect**									
	REx → IU					0.019	0.673		0.000
	REx → PH					0.158***	4.621		0.023
**Interaction**									
H5	SMA × REx → IU					0.058*	2.333		
H6	SMA × REx → PH					−0.032	0.991		

*p < 0.05, **p < 0.01, ***p < 0.001.

#### Model 2

**Figure 3:**
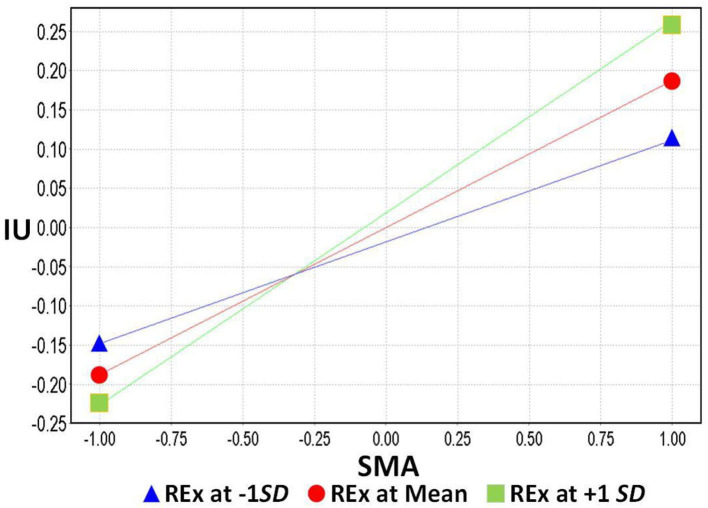
Moderating effect of regular exercise on social media addiction and internet use.

**Figure 4:**
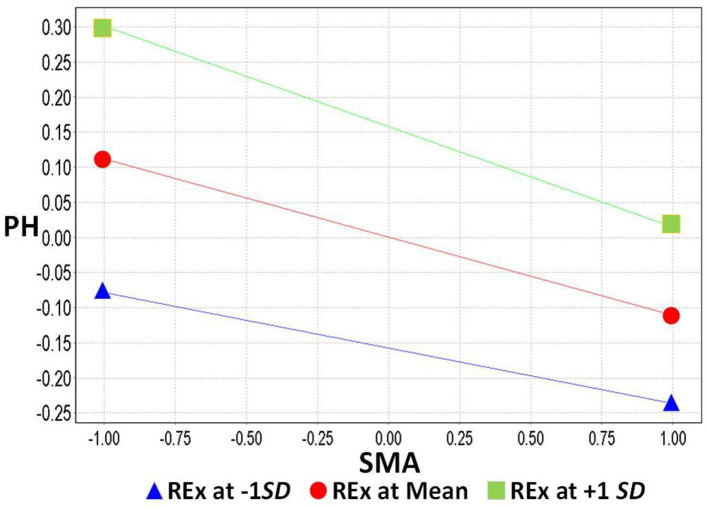
The moderating effect of regular exercise on social media addiction and perceived health.

In model 2, REx per week was included as a moderating effect to verify hypothesis 5 and how it influences the effect of SMA on IU. In addition, it examined the moderating effect of SMA on PH in hypothesis 6. H_5_: REx positively moderates the effect of SMA on IU. The standardized regression coefficient was 0.058 for the effect of REx on SMA and IU was significant (*t* = 2.333, *p* = 0.020 < 0.05); therefore, it is assumed that the effect of H_5_ moderation was valid and positive, as can be seen in Figure 3. On the other hand, in H_6_: the standardized regression coefficient of REx on SMA and PH was −0.032, which was not significant (*t* = 0.991, *p* = 0.322 > 0.05), so the hypothesis of H6 moderation was not valid in this study, as can be seen in Figures 2–4, and Table 6.

## Discussion

This study was conducted to investigate how REx interventions moderate the effect of SMA on IU and PH. This study constructed a research model based on Maslow's hierarchy of needs and the Person Environment fit theory, and employed a secondary data research method using data from the 2019 “Survey Research Data Archive,” Vol. 7, No. 5, with adult citizens aged 18 years or older in Taiwan. It has been known that physical function and health gradually decline with age, and that different generations of people, such as young, old, and older adults, have different habits of using the Internet. In other words, the results obtained in this study were more accurate after excluding the effect of age.

Based on the theory of Person Environment fit, this research successfully constructed a model of SMA on PH in adults. SMA will positively affect IU. When social media addiction increases by 1 unit, it will positively increase the use of the Internet by ~0.19 units. This result is consistent with previous studies (17–20). Moreover, 1 unit of SMA has a negative impact on PH of about 0.13 units, which is also consistent with previous research findings (19, 26).

Although many studies in the past have pointed out that health problems are associated with increased IU (9, 17, 27), there are also a number of studies that have suggested that social media addiction has many adverse health effects (19, 26). However, in this study there were no statistically significant effects on the health effects of IU, but the effects of IU are both immediate and caused by prolonged sedentary and inactivity, where health problems such as eye fatigue, anger, and sleep disturbances (75) can be improved immediately through a period of time. For IU to have a more significant and serious impact on health, a longer period of sedentary and inactive lifestyle is required (20), and such slow changes over a long period of time may not be clearly sensed by self-perceived health at times. Therefore, the health effects of IU alone may not be as direct. In addition, this study also found that there was no statistically significant effect of SMA on PH mediated by IU; perhaps this is related to the fact that there are many factors that affect physical health, and SMA affects a wide range of health dimensions.

Numerous studies have pointed out that there are many health benefits from performing regular exercise (28) and some scholars have suggested that regular exercise can reduce the health effects of social media addiction (29–32). However, in the empirical data of this study, regular exercise did not moderate the health effects of SMA; this result does not negate the health benefits of regular exercise, but simply means that there is no interaction between REx and SMA. In other words, the health effects of SMA and REx are two separate and parallel relationships. It is interesting to note that one can bring health benefits while the other is a loss of health, but the two do not affect each other.

Finally, in the past, most scholars thought that the regulation movement could reduce the use of IU behavior in SMA, which is a one-way all-or-nothing effect, but it is interesting to find that the effect may not be the same as we thought it would be. An interesting finding of this study was that REx moderates SMA and has a negative effect on the relationship between SMA and IU. This subtle change in direction varies in the degree of SMA and helps us to further understand the possibilities of regular movement intervention strategies. Therefore, when people's SMA is low (lower than average), regular exercise can indeed moderate the reduction of IU behavior; however, when SMA exceeds the threshold, the effect will be reversed, and the more regular exercise people have, the more they will use the Internet. This may be due to the fact that individuals with SMA have an uncontrollable urge to go online, or monitoring social media for fear of missing messages, which is considered to be Compulsive Internet Behavior (76, 93). The structure of social media is based on the characteristics of users' sharing and participation behaviors. In order to increase their own media content and attract others' attention, social media users engage in conspicuous behaviors, frequently using and disclosing information about their online selves (93). It seems that REx has become a topic of sharing for addicts, or is used for online check-ins to increase the buzz, thus creating the phenomenon that the more you exercise, the more you use the internet.

## Conclusion

### Theoretical Implications

The present study successfully applied the Person Environment fit theory to construct a model of the health effects of SMA in adults, which has been more commonly applied in organizational behavior and industrial management. In addition, the study applied Maslow's hierarchy of needs theory, which states that in addition to internally motivated social needs, people also engage in organizational expectancy behaviors in order to integrate into an organization or group. In the diverse and increasingly complex environment of postmodernism, this theory can be used to explore the behaviors of more individuals in different environments due to environmental influences.

### Practical Implications

SMA and excessive IU do pose health concerns in a modern society with advanced Internet technology, and REx can indeed bring many health benefits. Therefore, in using REx as a behavior change strategy to improve the health effects of SMA, first of all, it is necessary to establish a correct concept of exercise and a clear goal, and the planned exercise prescription should be in line with the FITT (Frequency, Intensity, Time, Type) principle of exercise training. Secondly, different intervention strategies should be given to different levels of addiction. For example, for low social media addiction, direct application of appropriate regulation of sports can reduce their internet usage behavior. For people with deeper SMA, REx alone may not be enough, and other psychological support or behavior change strategies are needed to reduce their IU. Finally, based on the theory of Person Environment fit, if sports and online virtual communities are combined to form a sports online community to create a virtual sports environment, using the characteristics of active sharing and active participation of social media will help REx become one of the main concerns of the community. However, in this circumstance, it may be one of the solutions that can transform the harm of social media into a powerful positive impact on health.

### Limitations and Future Study

Although the results of this study are valuable, there are some limitations that should be noted. The main research limitations are described as follows: First, from the perspective of exercise, each exercise must reach a fixed time and intensity in order for the individual to produce exercise benefits. This study used the frequency of regular exercise per week as a judgment of the amount of exercise, but there are still differences due to the different feelings and perceptions of each individual. Therefore, it is necessary to infer that the effects of all exercise types have their limitations. Second, since there are many types of social media and Internet usage behaviors, this study only selected the more common social media and Internet usage behaviors as observation variables. Third, although PH is a convenient tool for measuring one's own health status, it cannot achieve the accuracy of physiological indicators, especially in physiological sub-health states, but psychological cognition may not be able to feel the physiological changes, so there are still limitations when inferring the true health of an individual.

This study uses secondary data and official survey data, and the sampling is in line with the population distribution in Taiwan, even if it is low, there is still room for improvement. However, the research results are somewhat representative of the current social network usage of adults over the age of 18 in Taiwan. In addition, *R*^2^ has led us to discover that SMA may not account for as many of the health factors that affect the general population as one might think. Therefore, there may be other influential factors that have not been included in the representation; thus, future studies may expand the research model by adding other factors. This study found that the directional nature of REx to promote or reduce online behaviors in people with different levels of SMA varies, but to what extent SMA is considered high and to what extent it is considered low remains to be further studied. Finally, wearable devices have become popular in recent years for behavioral measurements such as regular exercise measurements, daily sleep hours, and so on. Future studies may implement wearable devices to record or monitor exercise more accurately to further clarify the relationship between REx, SMA, and PH.

## Data Availability Statement

The datasets presented in this study can be found in online repositories. The names of the repository/repositories and accession number(s) can be found at: doi: 10.6141/TW-SRDA-C00351_2-1.

## Author Contributions

All authors listed have made a substantial, direct, and intellectual contribution to the work and approved it for publication.

## Funding

This study was supported by the Ministry of Science and Technology, Taiwan (MOST 107-2627-H-003 -001 -MY5) and the Higher Education Deep Cultivation Project of National Taiwan Normal University (NTNU) sponsored by the Ministry of Education, Taiwan.

## Conflict of Interest

The authors declare that the research was conducted in the absence of any commercial or financial relationships that could be construed as a potential conflict of interest.

## Publisher's Note

All claims expressed in this article are solely those of the authors and do not necessarily represent those of their affiliated organizations, or those of the publisher, the editors and the reviewers. Any product that may be evaluated in this article, or claim that may be made by its manufacturer, is not guaranteed or endorsed by the publisher.
